# Calculated initial parenteral treatment of bacterial infections: Sepsis

**DOI:** 10.3205/id000053

**Published:** 2020-03-26

**Authors:** Klaus-Friedrich Bodmann, Rainer Höhl, Wolfgang Krüger, Beatrice Grabein, Wolfgang Graninger

**Affiliations:** 1 Klinik für Internistische Intensiv- und Notfallmedizin und Klinische Infektiologie, Klinikum Barnim GmbH, Werner Forßmann Krankenhaus, Eberswalde, Germany; 2 Institut für Klinikhygiene, Medizinische Mikrobiologie und Klinische Infektiologie, Klinikum Nürnberg, Germany; 3 Klinik für Anästhesiologie und Operative Intensivmedizin, Klinikum Konstanz, Germany; 4 Stabsstelle Klinische Mikrobiologie und Krankenhaushygiene, Klinikum der Universität München, Munich, Germany; 5 Vienna, Austria

## Abstract

This is the eleventh chapter of the guideline “Calculated initial parenteral treatment of bacterial infections in adults – update 2018” in the 2^nd^ updated version. The German guideline by the Paul-Ehrlich-Gesellschaft für Chemotherapie e.V. (PEG) has been translated to address an international audience.

Sepsis, defined as a life threatening organ dysfunction caused by a misregulated host response to an infection, is the third leading cause of death in Germany with a lethality rate of 30% to over 50%. An early, effective antimicrobial therapy is, next to infectious source control, the most important causal treatment option. It should be complemented by the mainly supportive measures of general intensive care therapy. Prior antimicrobial therapy, the patient’s medical history (e.g. risk factors for multiresistant agents) and small-scale epidemiology are to be considered as part of the therapeutic and practical decisions. A modification of the often needed broad initial calculated combination therapy is desirable. In the future, prompt measurements of plasma concentrations of antiinfectives, especially for the sepsis patient with diverse and partly conflicting pathophysiological changes, will have great importance regarding efficacy, toxicity and resistance development. In order to apply those complex strategies in clinical routine, there is a requirement for a strong interdisciplinary collaboration between the intensive care unit, clinical infectiology, microbiology, and clinical pharmacology, ideally in the framework of a functional antimicrobial stewardship program.

## Zusammenfassung

Dies ist das elfte Kapitel der von der Paul-Ehrlich-Gesellschaft für Chemotherapie e.V. (PEG) herausgegebenen S2k Leitlinie „Kalkulierte parenterale Initialtherapie bakterieller Erkrankungen bei Erwachsenen – Update 2018“ in der 2. aktualisierten Fassung.

Sepsis als die dritthäufigste Todesursache in Deutschland mit einer Letalität von 30 bis über 50% ist definiert als lebensbedrohliche Organdysfunktion, die durch eine fehlregulierte Wirtsantwort auf eine Infektion hervorgerufen wird. Die frühe, wirksame antimikrobielle Therapie stellt neben der Fokussanierung/-kontrolle die wichtigste kausale Behandlungsoption dar, ergänzt durch die allgemeine Intensivtherapie mit ihren vor allem supportiven Maßnahmen. Eine antimikrobielle Vortherapie, die Vorgeschichte des Patienten (z.B. Risikofaktoren für multiresistente Erreger) und die Kleinraumepidemiologie sollten unbedingt in die therapeutischen und praktischen Erwägungen einbezogen werden. Eine Modifizierung der zunächst oft breit notwendigen kalkulierten Kombinationstherapie ist anzustreben. In Zukunft wird der zeitnahen Plasmakonzentrationsbestimmung von Antiinfektiva gerade beim Sepsis-Patienten mit seinen vielfältigen, teils gegenläufigen pathophysiologischen Veränderungen eine herausragende Bedeutung im Hinblick auf Wirksamkeit, Toxizität und Resistenzentwicklung zukommen. Um diese komplexen Strategien im klinischen Alltag erfolgreich umsetzen zu können, bedarf es der engen Zusammenarbeit des Intensivmediziners/Klinikers mit der klinischen Infektiologie, der Mikrobiologie und der klinischen Pharmakologie, idealerweise im Rahmen eines funktionierenden Antimicrobial Stewardship Programmes.

## Introduction

The treatment of sepsis, especially with increasingly multidrug-resistant or selective pathogens (acronym “ESCAPE” [1]), represents one of the greatest challenges for clinically active physicians [2], [3].

Particularly in the field of intensive care, sepsis and septic shock are of particular importance due to increasing incidence, a slight drop in mortality of 30–50% and high costs.

In 2013, according to a survey by Fleischmann et al. [4] 279,530 cases of sepsis were reported to the Institute for the Hospital Remuneration System (InEK), with 67,849 of these patients (24.3%) dying. The mortality rate appears to be particularly high in patients with severe sepsis (60.3%). Sepsis is the third leading cause of death in Germany.

These data are complemented by a prospective, multicenter point-prevalence study (INSEP Study), in which 11,883 patients from 133 German intensive care units were investigated. Of these, 1,503 patients (12.6%) had a diagnosis of severe sepsis or septic shock, of which 860 cases (57.2%) were of nosocomial origin. The mortality of patients with sepsis was 34.3% during intensive care stay, compared to 6% of patients without sepsis. Overall, this study confirmed the tendency towards slightly lower mortality compared to previous studies but increasing prevalence of sepsis [5].

International comparisons of the incidence and mortality of sepsis are difficult as disease patterns, age structures, epidemiologically available data, and criteria for hospitalization or intensive care units differ significantly between countries. With these limitations, the mortality of severe sepsis is estimated at an average of 28% [6].

Fungal infections must be a consideration in patients in non-neutropenic intensive care. In the German prevalence study [7], fungi were detected microbiologically as the cause of severe sepsis in 17.8% of cases. In the US *Candida* spp. are now the third most frequent pathogen in blood cultures from patients in intensive care units [8], in Germany the fourth most frequent with the highest pathogen-associated mortality [9].

The predisposing diseases of sepsis include all forms of immune deficiency, such as tumors, diabetes mellitus, kidney and liver diseases and hemoblastosis, in the field of surgical intensive care for example polytrauma, burns and major high-risk procedures such as organ transplants. Effective antimicrobial treatment is the most important causal treatment option in addition to early source control. It is complemented by general intensive care with its mainly supportive measures [10].

According to current knowledge, Schuster’s definition of microbial sepsis is still considered the best [11]: “Sepsis is the sum of the life-threatening clinical manifestations and pathophysiological changes in response to activity of pathogens and their products entering the bloodstream from a focal point of infection, activating large biological cascade systems and specialized cell systems and triggering the formation and release of humoral and cellular mediators.”

The current criteria for the diagnosis of sepsis consist of the detection of an infection and at least two of the following four criteria [12]:

1. Fever above 38°C or in rare cases hypothermia below 36°C
2. Tachypnea above 20/min or hypocapnia with a PaCO_2_ <32 mm Hg
3. Tachycardia above 90/min
4. Leukocytosis greater than 12,000/mm^3^ or leukopenia less than 4,000/mm^3^ or normal white blood cell count left shift in differential blood count (more than 10% immature forms)

The reduction to these four “SIRS criteria” or the necessity of ≥2 of the criteria was not uncontroversial because on the one hand up to a quarter of cases of sepsis are not covered by them and on the other hand SIRS criteria have been met even by simple, uncomplicated infections [13], [14], [15]. Septic conditions were classified into different degrees of clinical severity according to the old American consensus definition: SIRS, sepsis, severe sepsis, septic shock.

**Figure 1:**
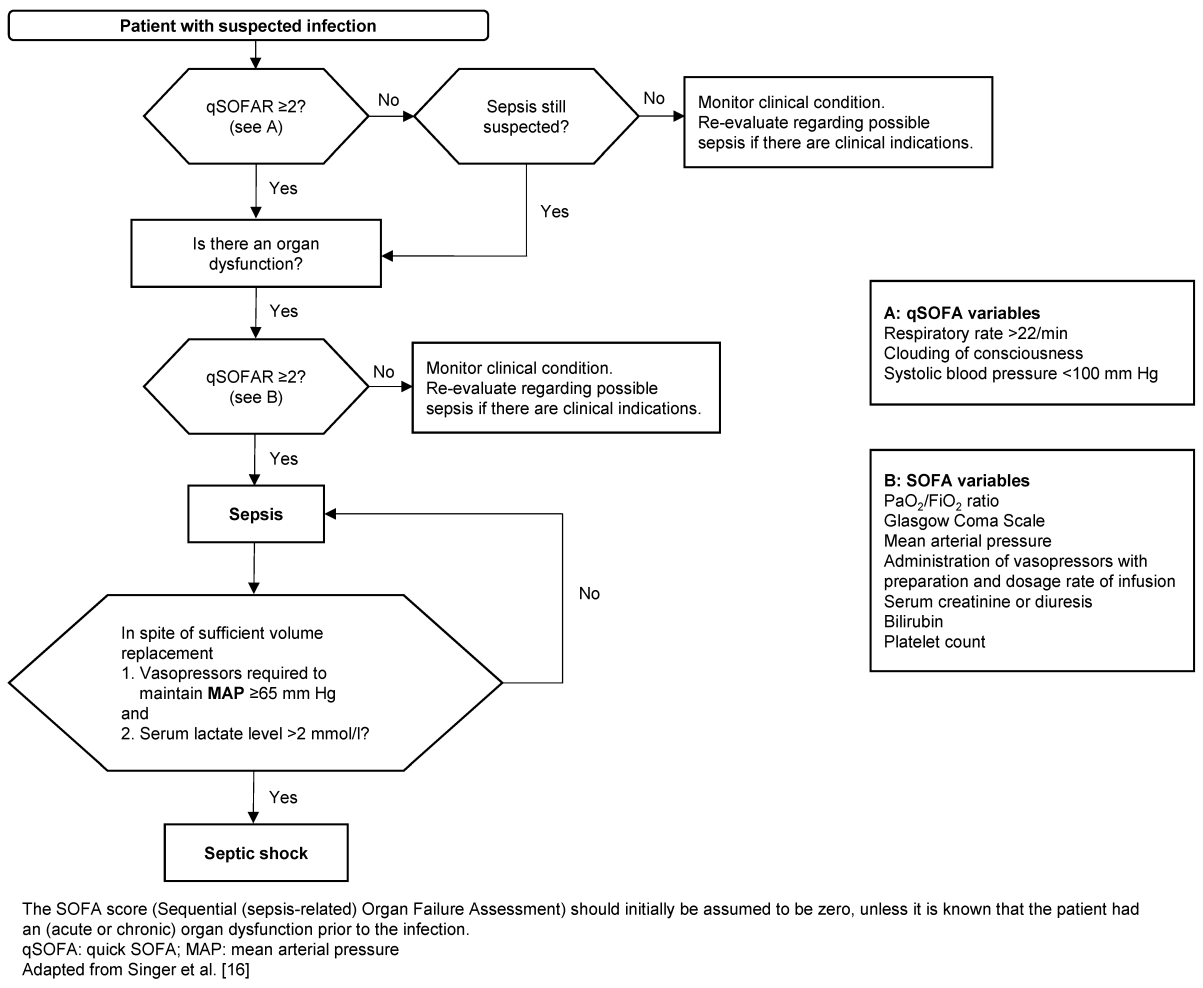
Flowchart to identify patients with sepsis and septic shock

A task force of 19 experts has revised the definition of sepsis on behalf of the two world-leading societies ESICM (European Society of Intensive Care Medicine) and SCCM (Society of Critical Care Medicine), which is now called “Sepsis-3” [16], [17], [18]. According to this new definition, sepsis is defined as a “life threatening organ dysfunction caused by a misregulated host response to infection”, i.e. the new “sepsis” is the old “severe sepsis”. The focus is now on the SOFA score; the SIRS criteria on the systemic inflammatory response of the body have been dropped. A “qSOFA” (quick SOFA) is intended to facilitate screening without lab testing in non-intensive care patients (Figure 1 (Fig. 1)):

- Respiratory rate ≥22/min
- Altered consciousness (GCS <15)
- Systolic blood pressure ≤100 mm Hg

qSOFA is considered positive if ≥2 criteria are met: follow by further search for organ dysfunction (SOFA score), start or escalate treatment, intensify monitoring.

Organ dysfunction is defined as an acute change of the SOFA score ≥2 points as a result of the infection (mortality ≥10%). The baseline SOFA score is assumed to be zero in patients with no known organ dysfunction. The SOFA score parameters are respiration, coagulation, liver, cardiovascular system, CNS, kidney.

Septic shock is defined as: Sepsis + vasopressor administration required to maintain a mean arterial blood pressure ≥65 mm Hg in persistent hypotension + serum lactate >2 mmol/l (>18 mg/dl) despite adequate volume replacement. For septic shock, hospital mortality exceeds 40%. 

The pathophysiological findings of recent years show that septic conditions are caused by a complicated network of pro- and anti-inflammatory cytokines [19], [20]. A sepsis event is a dynamic process of transition from the stage of “simple sepsis” to “severe sepsis” or “septic shock” with organ dysfunction or organ failure but also the development of septic organ colonization. A detailed description of intensive care supportive and adjunctive treatment measures would go far beyond the objectives of these guidelines on calculated anti-infective initial treatment. For this we refer to the current guidelines of the Surviving Sepsis Campaign [21]. 

Rapid, adequate antimicrobial treatment and, whenever possible, early source control in the first few hours is key to patient survival. In a retrospective study from 2006 [22] Kumar was able to show that with every hour of treatment delay after onset of hypotension in septic shock mortality increases by 7.6%. To some extent conflicting studies were published on this topic in the following years. A recent meta-analysis [23] seems to suggest that there is no benefit regarding mortality if antibiotics are administered in sepsis within the first 3 hours after initial assessment in the emergency department or one hour after the onset of septic shock. In addition to methodological weaknesses (7 studies could not be included due to failure to communicate with the authors), no single randomized, controlled study was included because there were none. In addition, the studies were not limited to those with adequate, effective treatment and no statement was made on multiresistant pathogens or on source control. 

In the current guidelines of the Surviving Sepsis Campaign, a strong recommendation is made for giving intravenous antibiotics no later than one hour after the diagnosis of sepsis or septic shock, although the evidence for this procedure is considered moderate [21]. This is supported by other current data [24], also from Germany [25]. 

Due to the increase in multidrug-resistant pathogens (especially MRSA, VRE but especially *Acinetobacter* *baumannii*, *Pseudomonas* *aeruginosa* and Enterobacteriaceae type 3MRGN and 4MRGN) [26] it is often necessary to start broad or even combined, antimicrobial treatment to adequately cover the pathogen spectrum [27]. Prior antimicrobial treatment and the patient’s history (for example risk factors for MRGN pathogens) should necessarily be included in the therapeutic and practical considerations (such as isolation).

The need for antimicrobial treatment should be reconsidered [28] and re-evaluated [29] daily. Combination treatment should be de-escalated once microbiological findings are available (less broad, discontinuation of a combination partner) [30].

In the physiologically and pharmacologically complex situation of sepsis, it is recommended to treat patients with high doses initially (i.e. in the first few days) in order to quickly reach a sufficiently effective level in sepsis patients with a high volume of distribution and creatinine clearance which is often elevated in hyperdynamic circulatory situations [31]. However, there are few data with good evidence on this topic. Attention should always be paid to signs of antimicrobial toxicity and possible interactions. Subsequently, the dosage should be adjusted to the organ deficiencies (kidney, liver). In the future, determination of plasma concentrations of antibiotics and antimycotics will be particularly important in sepsis patients in terms of efficacy, toxicity and development of resistance [31], [32], [33], [34], [35].

According to the current state of knowledge, in order to manage treatment, especially regarding the question of termination and effectiveness of antibiotic treatment, repeated determination of procalcitonin (PCT) in the serum should be carried out in addition to clinical assessment [22], [36], [37], [38], [39].

In order to be able to successfully implement the above-mentioned strategies in everyday clinical practice, it is necessary for intensive care physicians and clinicians to work closely with clinical infectiology, microbiology and clinical pharmacology. The modern term “antimicrobial stewardship” [40], [41] describes this approach. For example, infectiological advice for *Staphylococcus* *aureus* bacteremia significantly increased the quality of treatment and decreased mortality and length of hospital stay [42].

Overall, sepsis is a heterogeneous disease which is difficult to diagnose in the early stages and difficult to treat in the late stages. Early intervention improves the prognosis. Rapid and adequate antimicrobial therapy, microbiological diagnostics, source control and supportive treatment of internal organ dysfunction are the cornerstones of successful sepsis treatment. Nevertheless, excessive antimicrobial treatment must be avoided because of the expected collateral damage [43].

## Microbiology and current resistance situation

The current recommendations on blood culture diagnostics were published within the scope of the “MiQ guidelines” (Quality standards in microbiological-infectiological diagnostics of the German Society for Hygiene and Microbiology, DGHM). This contains instructions on the collection of blood cultures, the place of collection, the procedure for venipuncture and for sample transport and processing, with and without an automatic detection system. When taking blood cultures, if possible before initiating antibiotic treatment, the following points should be observed in particular:

- fresh puncture of a peripheral vein, take samples from existing catheters only in addition
- hygienic hand disinfection
- wipe or spray disinfect the skin on an area of at least 5x 5 cm with alcoholic disinfectant, exposure time 1 min.
- second skin disinfection inside out with sterile swab
- wear disposable gloves
- no re-palpation of the puncture site
- venipuncture and removal of 8–10 ml (5–10 ml) of blood per blood culture bottle, i.e. 16–20 ml per blood culture set
- taking three blood culture sets
- wipe septum tops of blood culture bottles with alcoholic disinfectant
- wait for the disinfectant to dry
- inoculate blood culture bottles with fresh cannula (not used!) or use closed sampling system (TRBA!)
- do not aerate the aerobic bottle
- immediately transport blood culture bottles to the lab

The spectrum of sepsis pathogens is broad. In the German SEPNET study, 55% of cases were caused by Gram-positive bacteria, 54% by Gram-negative microorganisms and almost 18% by *Candida* species. Their sum exceeding 100% is explained by polymicrobial infections [7].

Data on the resistance situation in blood culture isolates in Germany are available from the ARS Antibiotic Resistance Surveillance System from 2015 [44] (see also chapter 2 [45]).

The proportion of methicillin-resistant strains of *Staphylococcus* *aureus* has fallen slightly in recent years, reaching 11.8% (n=7,740). However the proportion of methicillin-resistant isolates in coagulase-negative staphylococci remains high at 58.8% (n=27.804). The proportion of glycopeptide-resistant *Enterococcus* *faecium* strains in blood culture isolates stood at 12.2% in 2015 (n=1,729), falling slightly in 2011 from a previous high of 14.8% (n=573).

In the case of *Escherichia* *coli*, the proportion of fluoroquinolone-resistant strains has fallen slightly in recent years and for ciprofloxacin was 20.7% (n=11,611). The proportion of cefotaxime-resistant isolates expressing the presence of an ESBL is currently 11.5% (n=9,958). In Klebsiella pneumoniae, the ciprofloxacin resistance rate has remained more or less constant in recent years, reaching 12.1% in 2015 (n=2,051). The rate of ESBL-formers in *Klebsiella* *pneumoniae*, again measured by cefotaxime resistance, has also been virtually unchanged in recent years and last year was 13.0% (n=1,796). In the meantime, the first carbapenem-resistant Klebsiella isolates have been detected in blood cultures, even though the proportion of 0.2% (meropenem, n=2,032) is still very low.

In the case of *Pseudomonas* *aeruginosa*, the resistance rate to ceftazidime is 9.1% (n=10,769), to piperacillin/tazobactam 15.6% (n=1,073) and to meropenem 8.1% (n=1,081) but if intermediate strains are added to meropenem, this results in 16.7% intermediate and resistant isolates.

## Pharmacokinetics and pharmacodynamics

The pharmacokinetics and pharmacodynamics of antibiotics in patients with severe sepsis and septic shock are sometimes significantly different from data collected in less severely ill patients. Pharmacokinetics are influenced by complex, sometimes counterproductive processes, so that antibiotic levels are difficult to predict. In the early stages of sepsis, hyperdynamic circulatory conditions dominate in many patients, leading to increased clearance of renally eliminated anti-infective agents (augmented renal clearance, ARC) compared to healthy people. The capillary leak also causes expansion of the extracellular space. These two factors lead to unexpectedly low plasma levels of hydrophilic and renally eliminated antibiotics, affecting most beta-lactams and also aminoglycosides and vancomycin [46], [47], [48]. As a result, therapeutic drug monitoring (TDM) should be carried out for these antibiotics, which is mandatory for aminoglycosides and vancomycin in any case because of their high toxic potential [49], [50]. For beta-lactams TDM would also be useful but it is rarely available for routine clinical work. Changes in pharmacokinetics are less pronounced for antibiotics with large volumes of distribution (for example fluoroquinolones), i.e. with predominantly intracellular accumulation [46].

As a progressing sepsis leads to more and more organ dysfunction, especially with renal insufficiency, reduced elimination leads to increased plasma levels and possibly the accumulation of mostly ineffective but potentially toxic metabolites of the drugs [47]. Added to this, for antibiotics with high protein binding, is displacement from binding through other drugs or due to pH shifts . If alternatives exist, consideration should therefore be given to antibiotics with lower protein binding and low toxic potential (for example in MSSA sepsis a cephalosporin instead of flucloxacillin, which is more than 90% protein-bound and has a high hepatotoxic risk).

There is however no answer to the question of whether the percentages given for protein binding can be transferred to the situation of treating critically ill patients. There is a discussion of whether it would not be better for the plasma levels of beta-lactam antibiotics in critically ill patients to be above the MIC during the entire dosing interval. In addition, it is stated that plasma levels should be up to 4 times above the MIC to ensure tissue penetration of the antibiotics. However in many cases this would mean a significantly higher dosage than previously used for beta-lactam antibiotics [32].

After initial administration of a loading dose to rapidly achieve the required effective level, continuous infusions of beta-lactam antibiotics, especially for intermediate-susceptibility pathogens, could improve treatment outcomes in critically ill patients. However in addition to practicability (shelf-life at room temperature, incompatibility with other drugs), the use of a continuous infusion without therapeutic drug monitoring (TDM) carries the risk that the plasma levels may be permanently below the MIC of the (often unknown) pathogen. Continuous antibiotic infusion should therefore only be used if TDM is available promptly, ideally complemented by determination of the MIC of the antibiotic for the pathogen (see chapter 3 [51]).

If TDM is not available, prolonged infusion of beta-lactams over 3–4 hours is a possible sensible compromise. In this way, the disadvantage of short infusion with (unnecessarily) high peak levels and rapid drop of the effective level below the MIC is avoided as well as the potential danger associated with continuous infusion of permanently staying below the MIC. For rapidly reaching a therapeutically effective level, the initial dose should be given in the form of a traditional short infusion. 

In order to clinically translate the insights into the peculiarities of pharmacokinetics and pharmacodynamics, TDM should be established for the most important beta-lactams used at the hospital in question in patients with severe sepsis (for example ceftazidime or cefepime, piperacillin/tazobactam, meropenem or imipenem). Without TDM, the use of continuous infusions is discouraged.

In some hospitals where TDM is not available, it is common practice to use higher doses for patients in the hyperdynamic phase of sepsis if the kidneys are still functioning, at least on the first day of treatment. Treatment may then be to administer the initial dose of the antibiotics mentioned above, when indicated, as a short infusion, followed by prolonged infusions at the usual times on the ward. It should be pointed out that only Doripenem – which was withdrawn from the market – was approved for prolonged infusion. For the other beta-lactams there are promising individual studies and meta-analyzes demonstrating better clinical efficacy of continuous or prolonged infusion. In addition to a few prospective studies, the meta-analyzes also include retrospective and cohort studies with limited significance [52], [53]. A recent meta-analysis [54] evaluated randomized, prospective studies of continuous versus intermittent beta-lactam infusion based on individual patient data [55], [56], [57]. There was a significant reduction in hospital mortality with continuous infusion (19.6% versus 26.3%) but without therapeutic drug monitoring [54].

Despite convincing data in vitro and in vivo, it remains difficult to demonstrate the superiority of continuous or prolonged infusions in clinical trials. The reasons for this are manifold, ranging from the difficulty of infection diagnosis to lack of pathogen identification to the fact that, in very sensitive pathogens, effective levels above the MIC can be achieved for a sufficient length of time even with traditional intermittent bolus administration [58], [59], [60].

In terms of pharmacodynamics, aminoglycosides are a mirror image to beta-lactams. This is because the bactericidal effect is improved by high peak levels, followed by pronounced post-antibiotic effects, which allow the plasma levels to drop below the MIC for many hours. TDM is established nationwide and is obligatory due to the high nephrotoxicity and ototoxicity. After bolus administration of the total daily dose, the next dose is given at the earliest after 24 hours, when the trough level for gentamicin or tobramycin is below 1 mg/l. 

For fluoroquinolones, in order to optimize efficacy, it is recommended to generate the largest possible area of the time-plasma-mirror curve above the MIC (AUC>MIC). In clinical practice, this complicated mathematical term is of little use. Due to the mathematical link, fluoroquinolones can be better understood as analogous to aminoglycosides as peak-level-dependent antibiotics [47].

## Treatment recommendations

In almost all patients, the initial, antimicrobial treatment is calculated according to the intervention treatment recommended by the Paul-Ehrlich-Gesellschaft. In some of the patients it is possible to modify initial intervention treatment through detecting the pathogen with an antibiogram. Initial anti-infective selection is influenced by the suspected source of infection, underlying diseases and risk factors (for example whether an infection is community-acquired or nosocomial, time of onset of infection and prior antimicrobial treatment).

Table 1 (Tab. 1) shows treatment recommendations for unknown pathogens in relation to the type and localization of the infection and Table 2 (Tab. 2) shows treatment recommendations for cases where the pathogens have been identified. Table 3 (Tab. 3) shows the recommendation grades for the use of antibiotics in the indication “nosocomially acquired sepsis with unknown pathogen and unknown site of infection”. The wide variety of treatment options listed in Table 1 (Tab. 1) and Table 2 (Tab. 2) is due to different degrees of severity of the disease and the risk factors of the patient. The duration of treatment should be 7–10 days. Exceptions are slow response to treatment, a non-restorable focus and immunosuppression [4]. In PCT-directed antibiotic treatment, the duration of antibiotic treatment may also be shorter than 7 days if by that time there has been a PCT decrease of more than 80% compared to the highest measured value or if the absolute PCT has a measured value ≤0.25 ng/l. 

Although the data are insufficient, initial combination treatment should always be performed in patients suffering from life-threatening illness (Table 1 (Tab. 1)). This approach is supported, amongst other things, by the results of the Surviving Sepsis Campaign. Dellinger et al. recommend administration of one or more substances with a broad spectrum and good penetration into tissue for calculated initial treatment [28].

This strategy should be evaluated after 72 hours at the latest. Combination treatment is explicitly called for in cases of suspected or proven *Pseudomonas* infection [28], [61]. Traditionally, aminoglycosides have been the preferred combination partners for beta-lactam antibiotics. The option of using fluoroquinolones as a combination partner of beta-lactam antibiotics is backed up by the work of Paul et al. [62], [63]. Fluoroquinolones offer pharmacokinetic benefits, are associated with lower toxicity, and there is no need to measure levels regularly. However, resistance rates for fluoroquinolones are consistently higher than for aminoglycosides. In view of the occasionally high fluoroquinolones resistance rates, fosfomycin is another option as a combination partner with good tissue penetration.

In cases of sepsis, all anti-infective drugs must be administered intravenously and in high doses. Neither sequential therapy nor dose reduction are proven by studies in this indication.

In severe sepsis or septic shock and with unknown sepsis focus, it should be combined with a lipopeptide (daptomycin) [7], [40], [64], [65] or a glycopeptide in high-risk patients with a high rate of MRSA. Alternatively, ceftobiprole (group 5 cephalosporin) may also be used in combination with a fluoroquinolone or fosfomycin in these patients since ceftobiprole has good efficacy against MRSA. However it is doubtful whether the approved dosage of 3x 500 mg i.v., in the form of a 2-hour infusion in patients with normal renal function, is adequate. According to the data published in ECCMID 2015 by Torres et al. [66] one should aim for a dose of 3x 1,000 mg ceftobiprole in such patients. Ceftolozane/tazobactam could also be a useful treatment option in this indication. Here, however, the lack of efficacy of this combination of substances against staphylococci and most anaerobes has to be considered. The dose for the treatment of sepsis should be 3x 3 g i.v.

In addition, anaerobes can be expected in sepsis which originates from the respiratory tract, especially with *Streptococcus* *pneumoniae*, *Staphylococcus* *aureus* and various Enterobacteriaceae as well as in aspiration pneumonia. In case of serious risk situations or hospital stays of more than 5 days, *Pseudomonas* *aeruginosa*, *Acinetobacter* spp. and *Stenotrophomonas* *maltophilia* can be expected. The pathogen spectrum can vary greatly from hospital to hospital. A recent study indicates that Gram-negative pathogens in ventilated patients can increasingly also be expected in short hospital stays [67]. In high-risk patients with severe sepsis or septic shock and high MRSA hospital rates, it should be combined with oxazolidinone (linezolid) [40]. Ceftobiprole, in combination with a fluoroquinolone or fosfomycin, is a useful alternative. Ceftobiprole is a treatment option that can also be administered at a suitable dose (3x 1 g i.v.) in cases of pneumogenic sepsis [66].

In addition, ceftolozane/tazobactam should be considered as a further option for calculated initial treatment in patients with severe sepsis or septic shock and unknown septic focus as well as in pneumogenic sepsis because of its excellent efficacy against pseudomonads (including MDR) and ESBL-producers. Its ineffectiveness against staphylococci and anaerobics must, however, be compensated by an appropriate combination partner.

If sepsis originates in the urinary tract without previous instrumental intervention, primarily *Escherichia* *coli* and *Proteus* *mirabilis* are to be expected as sepsis pathogens. After urological interventions other Enterobacteriaceae, *Pseudomonas* *aeruginosa*, enterococci and staphylococci must also be considered.

If the starting point is the intestine or a gynecological organ, the following pathogens must be expected: Enterobacteriaceae, anaerobes, enterococci, *Pseudomonas* spp., *Staphylococcus* *aureus*.

In biliary sepsis, pathogen colonization in the bile ducts and thus the risk of bacteremia increases with the degree of outflow obstruction. In occlusive ictus, more than 75% of patients have pathogens in their blood. The spectrum includes Enterobacteriaceae, enterococci and anaerobes. In post-operative bacteremia, cholangiotic sepsis and sub-hepatic abscesses as well as in interventional procedures (ERCP or endoscopic papillotomy), other Gram-negative pathogens, including *Pseudomonas* *aeruginosa*, have been identified. If sepsis originates from the gut/gynecological organs and biliary tract, in severe sepsis or septic shock, it can be combined with a glycylcycline (tigecycline) [40], [68], [69]. 

If the source is the skin or soft tissue, infections by *Streptococcus* *pyogenes*, *Staphylococcus* *aureus* (also MRSA) and mixed infections with the additional involvement of non-A streptococci, anaerobes, Enterobacteriaceae or *Pseudomonas* *aeruginosa* are possible.

The pathogen spectrum of catheter-associated sepsis includes coagulase-negative staphylococci, *Staphylococcus* *aureus*, Gram-negative rod bacteria, *Candida* spp., *Corynebacterium* *jeikeium* and propionibacteria. Another therapeutic option is the lipopeptide daptomycin [7], [65] as an alternative to the glycopeptide.

The monotherapy recommendations presented in Table 1 (Tab. 1) are based on the results of well-documented, randomized clinical studies. In contrast, there is a general lack of clinical studies on combination treatment recommendations. Accordingly, these recommendations are based on expert opinions, this is especially true for combination therapy with a fluoroquinolone.

## Note

This is the eleventh chapter of the guideline “Calculated initial parenteral treatment of bacterial infections in adults – update 2018” in the 2^nd^ updated version. The German guideline by the Paul-Ehrlich-Gesellschaft für Chemotherapie e.V. (PEG) has been translated to address an international audience.

## Competing interests

The authors declare that they have no competing interests.

## Figures and Tables

**Table 1:**
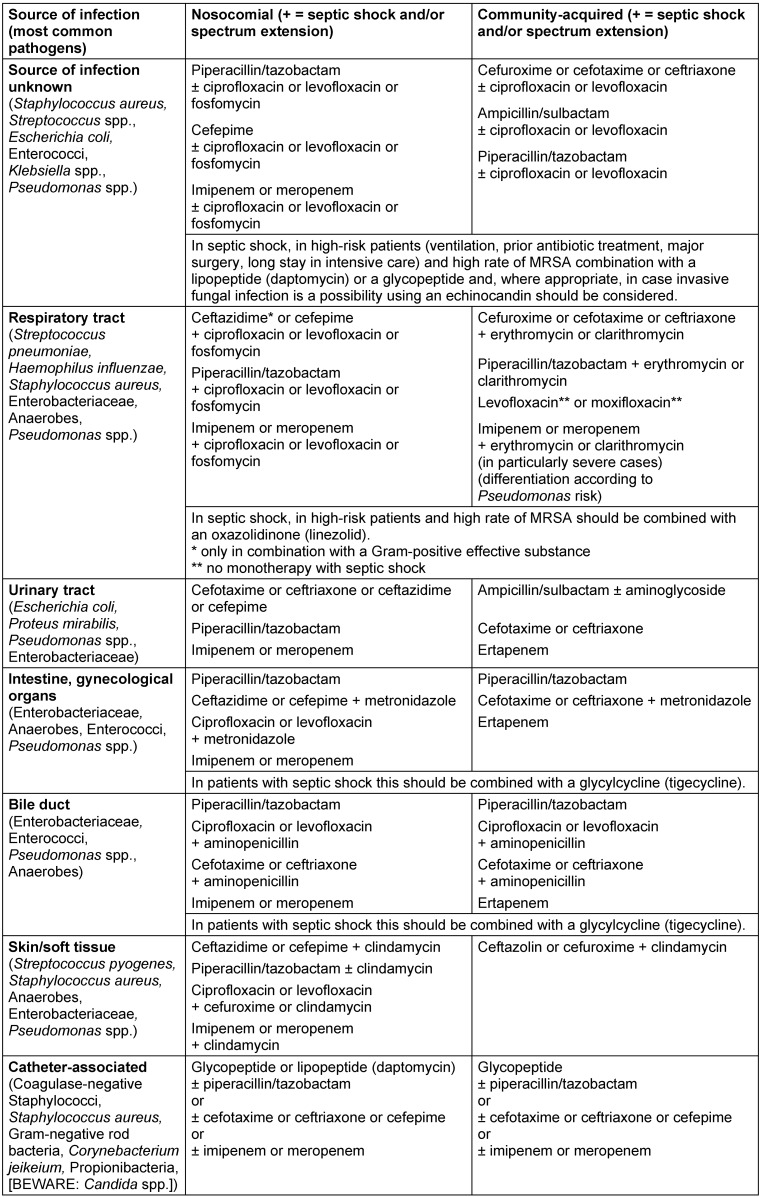
Recommendations for the treatment of sepsis with unknown pathogen. (Treatment recommendations are not intended for immunosuppressed and neutropenic patients.)

**Table 2:**
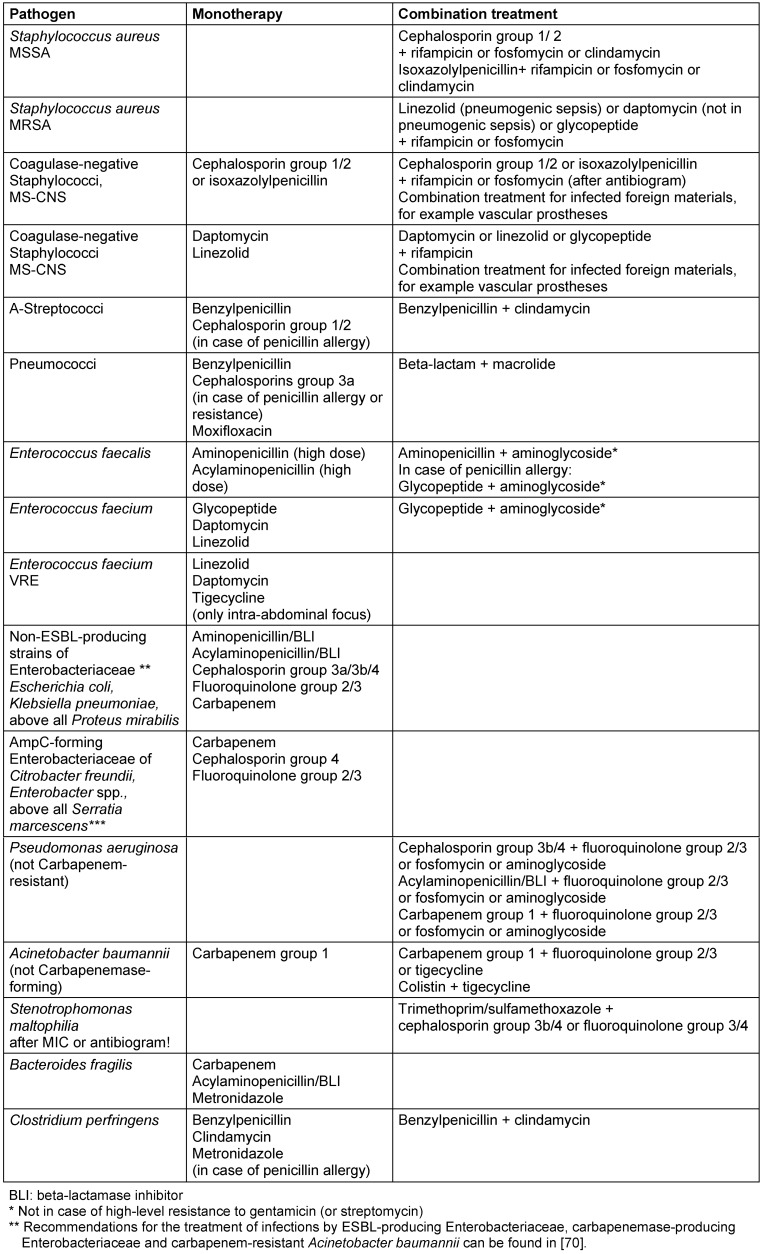
Recommendations for antibiotic treatment of sepsis where the pathogen is known

**Table 3:**
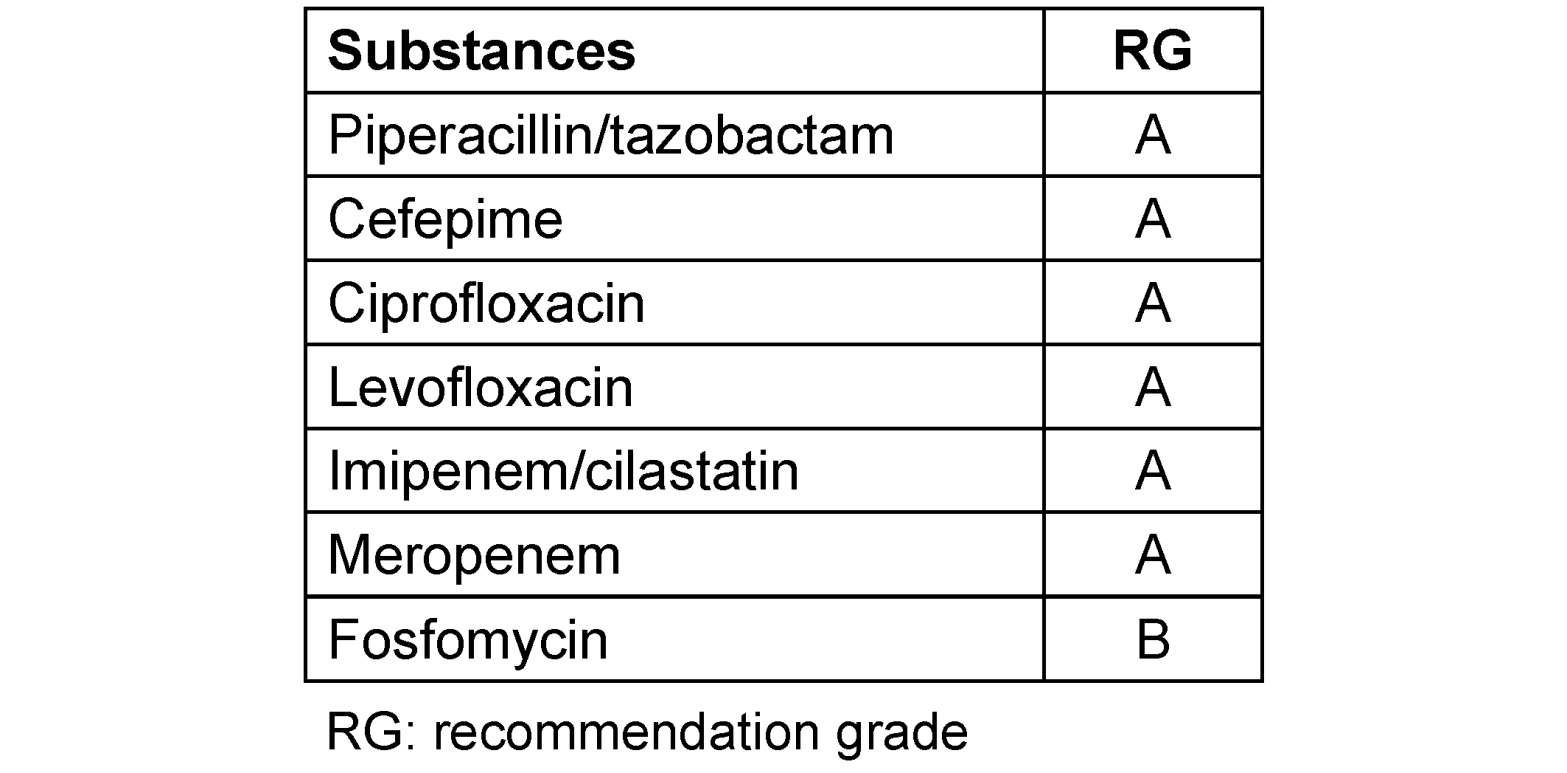
Recommendation grade for the use of antibiotics in the indication “nosocomial acquired sepsis with unknown pathogen and unknown site of infection”
